# Fronto-Parietal Brain Responses to Visuotactile Congruence in an Anatomical Reference Frame

**DOI:** 10.3389/fnhum.2018.00084

**Published:** 2018-03-05

**Authors:** Jakub Limanowski, Felix Blankenburg

**Affiliations:** ^1^Neurocomputation and Neuroimaging Unit, Department of Education and Psychology, Free University of Berlin, Berlin, Germany; ^2^Center for Cognitive Neuroscience Berlin, Free University of Berlin, Berlin, Germany; ^3^Wellcome Centre for Human Neuroimaging, University College London, London, United Kingdom

**Keywords:** body representation, multisensory integration, peripersonal space, rubber hand illusion, touch

## Abstract

Spatially and temporally congruent visuotactile stimulation of a fake hand together with one’s real hand may result in an illusory self-attribution of the fake hand. Although this illusion relies on a representation of the two touched body parts in external space, there is tentative evidence that, for the illusion to occur, the seen and felt touches also need to be congruent in an anatomical reference frame. We used functional magnetic resonance imaging and a somatotopical, virtual reality-based setup to isolate the neuronal basis of such a comparison. Participants’ index or little finger was synchronously touched with the index or little finger of a virtual hand, under congruent or incongruent orientations of the real and virtual hands. The left ventral premotor cortex responded significantly more strongly to visuotactile co-stimulation of the same versus different fingers of the virtual and real hand. Conversely, the left anterior intraparietal sulcus responded significantly more strongly to co-stimulation of different versus same fingers. Both responses were independent of hand orientation congruence and of spatial congruence of the visuotactile stimuli. Our results suggest that fronto-parietal areas previously associated with multisensory processing within peripersonal space and with tactile remapping evaluate the congruence of visuotactile stimulation on the body according to an anatomical reference frame.

## Introduction

When I see a body being touched and at the same time feel touch on the corresponding part of my skin, I know immediately that it is *my* body that I see being touched. If I felt the touch after observing it, or if I felt it on a different body part, I would very likely conclude that the body I see being touched is not mine. The brain constantly has to solve such causal inference problems when deciding whether two stimuli, registered by separate modalities, should be attributed to the same cause (i.e., “integrated” into one multisensory percept) or to different causes (Macaluso and Driver, 2005; Blanke, 2012; Ehrsson, 2012; Rohe and Noppeney, 2015; Kilteni et al., 2015; Samad et al., 2015). By nature, multisensory integration and the underlying causal inference are probabilistic, and inherently flexible. This is also the case for seen and felt touch, as demonstrated by the rubber hand illusion (RHI): when participants see a fake hand being touched and simultaneously feel a touch on the corresponding location of their real hand, they often report “feeling” the touch on the fake hand (Botvinick and Cohen, 1998). Crucially, the RHI only occurs if prior constraints of a pre-existing body model (an anatomically plausible shape and position of the fake hand) and basic rules of multisensory integration are satisfied—i.e., if the seen and felt touch occurs at the same time and at a corresponding location (Meredith and Stein, 1986; Makin et al., 2008; Tsakiris, 2010; Blanke, 2012; Ehrsson, 2012).

Brain imaging has established a link of the RHI to activity of the ventral premotor cortex (PMv) and the intraparietal sulcus (IPS, Ehrsson et al., 2004; Makin et al., 2007; Gentile et al., 2013; Limanowski and Blankenburg, 2015; Zeller et al., 2015). Research on non-human primates and, more recently, on humans has shown that the PMv and IPS contain neurons with visual and tactile receptive fields (Graziano, 1999; Graziano et al., 2000; Avillac et al., 2007; Gentile et al., 2011). The receptive fields of such visuotactile neurons are relatively large and often anchored to specific body parts, i.e., they respond to touch on that body part and to visual stimuli entering the space immediately surrounding it—the “peripersonal space” (PPS; Graziano et al., 1994; Rizzolatti et al., 1997; Graziano and Cooke, 2006; Brozzoli et al., 2012). A multisensory representation of the upper limbs and the PPS around them is thought to ultimately serve for action control, particularly for defending the body (Graziano and Cooke, 2006). The illusory change of the multisensory body representation induced by the RHI seems to be accompanied by a corresponding recalibration of PPS onto the rubber hand (Makin et al., 2008). It has hence been proposed that during the induction of the RHI, the PMv and IPS work together to integrate spatially and temporally congruent visual and tactile stimuli within the approximate limits of PPS anchored to the real hand, which after induction leads to a remapping of PPS and may even lead to an illusory self-attribution of the fake hand (Makin et al., 2008; Tsakiris, 2010; Blanke, 2012). In sum, a successful induction of the RHI mandates that the seen and felt touches occur on the same location of two (fake and real) correspondingly oriented body parts, within approximate limits defined by the PPS of the real hand prior to induction of the illusion.

However, when the body is touched, the stimulus is initially processed in a somatotopical, skin-based reference frame – implying the need for a “remapping” into external coordinate frames for comparison with information from other modalities such as with vision during the RHI (Yamamoto and Kitazawa, 2001; Azañón et al., 2010; Heed et al., 2015). Although the comparison processes underlying the RHI rely on a multisensory representation of the body (and the touch on it) in external space, there is also evidence for the involvement of a multisensory representation of the body’s structure that operates in an anatomical or homuncular reference frame (Haggard et al., 2006; Longo et al., 2010; Rusconi et al., 2014). Interestingly, in a behavioral study, Costantini and Haggard (2007) have shown that the RHI remained despite mismatches in the fake and real hands’ position, as long as stimulation was congruent in a “hand-centered spatial reference frame.” This indeed suggests that anatomically based comparisons may also determine the visuotactile integration process and the resulting self-attribution of the fake hand, and thus speaks to proposals of a distinct multisensory body representation according to an anatomical or homuncular reference frame. In functional imaging research on the RHI, however, the processing of visuotactile congruence in an anatomical reference frame has so far received little attention.

Therefore, we investigated human brain activity during visuotactile co-stimulation of a virtual hand and the real unseen hand at varying anatomical locations independently of the congruence of the hands’ orientations. We used a virtual reality-based experimental setup with stimulation locations on the index and little finger of the right hand, repeatedly varying both the virtual and real hands’ orientation, and controlling for spatial attention with a catch-trial task. This setup allowed us to isolate the effects of visuotactile congruence in an anatomical reference frame independent of visuoproprioceptive congruence and of external spatial visuotactile congruence. We stimulated the hands only briefly, because arguably the most interesting observations about the brain activity underlying the RHI can be made during the (usually several seconds long) “pre-illusion” period leading up to the illusion, during which the brain’s multisensory comparison and integration processes are most strongly engaged (Ehrsson et al., 2004; Limanowski and Blankenburg, 2015, 2017). We hypothesized that although tactile remapping itself is a fast process (Azañón et al., 2010; Heed et al., 2015), the decision of whether multisensory input during an attempted RHI induction should be integrated or not may as noted also rely on anatomical body representations (Haggard et al., 2006; Costantini and Haggard, 2007). We speculated that these visuotactile comparison and evaluation processes would engage the fronto-parietal brain areas previously implied in a multisensory representation of the PPS, i.e., the PMv and IPS.

## Materials and Methods

### Participants

Twenty healthy, right-handed volunteers (7 male, mean age = 28 years, range = 21–40, normal or corrected-to-normal vision) participated in the experiment, which was approved by the ethics committee of the Freie Universität Berlin and conducted in accordance with the approval.

### Experimental Design and Procedure

**FIGURE 1:**
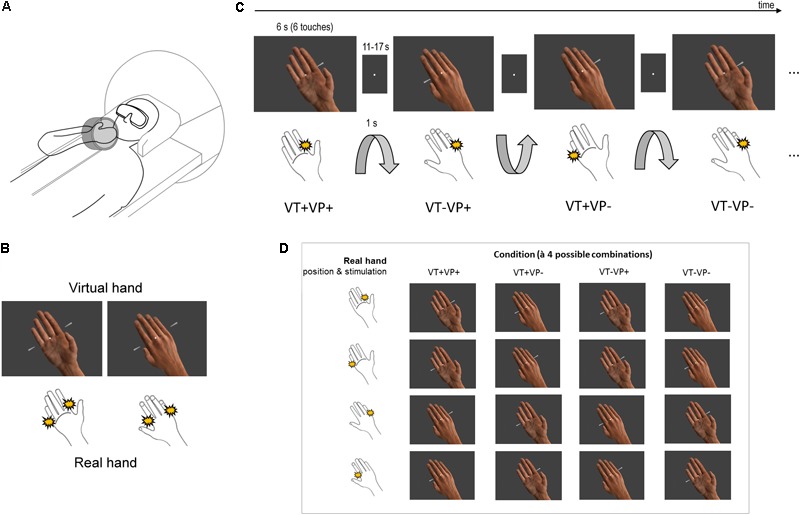
Experimental design. **(A)** Participants saw a photorealistic right virtual hand in either a palm or back facing orientation (presented in 3D via stereoscopic goggles), with their real unseen right hand placed above their chest, likewise palm or back facing. The orientation of the real unseen hand was changed by the experimenter in between conditions. **(B)** The virtual hand could be touched at the index or little finger by moving rods (both shown here for illustrative purpose). The participant simultaneously received electrotactile impulses at a corresponding location of the real index or little finger (both locations schematically indicated). **(C)** Schematic partial stimulus sequence. Between stimulation blocks, the orientation of the participant’s hand was passively switched (1 s rotation by the experimenter) between palm and back facing. During stimulation, the virtual hand was randomly displayed in a palm or back facing orientation (both orientations were anatomically plausible); visual and tactile stimulation locations were also random. Thus, each block (6 s, separated by an 11–17 s fixation-only baseline) was an unpredictable combination of touch applied to the same (VT+) or a different finger (VT–) of the real and virtual hand, under congruent (VP+) or incongruent (VP–) hand orientations. **(D)** Each of the 4 conditions consisted of 4 different combinations of touch locations and arm orientations.

During the experiment, participants lay inside the fMRI scanner with their right hand placed above their chest at a natural angle of about 45° and comfortably fixed in a custom foam-padded apparatus (**Figure 1A**, modified from Limanowski and Blankenburg, 2016a). The apparatus holding the hand could be rotated back-and-forth by the experimenter pulling on two nylon strings from outside of the scanner bore, so that either the palm or the back of the hand was facing the participant. We used stereoscopic goggles (VisuaSTIM, 800 × 600 pixels, 30° eye field) and the Blender graphics software package^1^ to present participants a photorealistic virtual hand in 3D in a similar, anatomically plausible position and location in space with respect to their real arm, with either the palm or the back of the virtual hand facing the participant. The real hand was always hidden from view by the goggles. The participant’s head was slightly tilted to align the perceived real and virtual hand locations as much as possible within the spatial constraints of the head coil, and participants were asked if the respective palm or back facing orientations of the virtual hand seemed to them plausibly aligned to their real unseen hand.

Participants fixated a white dot in the middle of the visual display throughout the entire experiment. During each 6 s long stimulation block, the virtual hand was presented in one of two orientations (palm or back facing the participant) and was touched by virtual rods at either the index or the little finger (**Figure 1B**). Simultaneously, the index or little finger of the participant’s real hand was stimulated by electrotactile impulses (200 μs long monophasic square wave pulses generated by a bipolar constant current stimulator, Digitimer DS7), delivered via MR-compatible adhesive electrodes attached to the respective finger’s first phalanx – the stimulation locations were constant throughout each stimulation block. The intensity, location, and timing of the electrotactile impulse was carefully adjusted for each participant before the scanning session, so that it matched the anatomical location where the touch was seen on the virtual hand, and felt synchronous with it; this was adjusted between the individual runs if necessary. Six such simultaneously seen and felt touches were delivered in each stimulation block (i.e., each 6 s long presentation of a particular combination), separated by a random inter-stimulus interval (0, 167, or 500 ms) to render the stimulation sequence unpredictable (**Figure 1C**). The stimulation blocks were separated by a randomly jittered 11–17 s fixation-only inter-block interval (IBI). During each IBI, 3–6 s (randomly jittered) after IBI onset, the experimenter swiftly rotated the participant’s hand from palm to back facing or vice versa while participants only saw the fixation dot; the rotation took 1 s and was announced and timed by auditory cues to the experimenter. To avoid surprise, the rotation was also announced to the participant by a brief disappearance of the fixation dot for 0.5 s prior to rotation onset.

The combination of touch locations and arm orientations resulted in 16 stimulation combinations, which were assigned to 4 conditions (à 4 stimulation combinations, **Figure 1D**): same finger touched, congruent hand orientations (VT+VP+); same finger touched, incongruent hand orientations (VT+VP-); different fingers touched, congruent hand orientations (VT-VP+); different fingers touched, incongruent hand orientations (VT-VP-). Each combination was presented twice per run in randomized order, resulting in 32 stimulation blocks and ∼11 min run length. Each participant completed 6 runs, with initial hand orientation counterbalanced across runs, and a practice session before scanning.

To control for attentional effects and to ensure constant fixation, we included a catch trial detection task. Throughout each run, the fixation dot unpredictably pulsated briefly (25% increase in size for 300 ms) 5–9 times. Participants had to report the number of pulsations verbally to the experimenter after each run.

After the scanning session, participants completed a brief questionnaire comprising the following two statements, rated on a 7-point scale from -3 (“do not agree at all”) to 3 (“fully agree”): “I was able to distinguish whether the orientation of the virtual hand and my real hand was congruent (corresponding) or incongruent (not corresponding)” and “It felt as if the virtual hand was my own hand”; the latter statement was rated separately for each of the four stimulation conditions. The (ordinally scaled) questionnaire data were evaluated using non-parametric Friedman and Wilcoxon tests. For comparisons between condition scores, we further report the common language (CL) effect size as the proportion of matched pairs for which the score in one condition is higher than the score in the other condition (Wuensch, 2015).

### fMRI Data Acquisition, Preprocessing, and Analysis

The fMRI data were recorded using a 3 T scanner (Tim Trio, Siemens, Germany), equipped with a 12-channel head coil. T2^∗^-weighted images were acquired using a gradient echo-planar imaging sequence (3 mm × 3 mm × 3 mm voxels, 20% gap, matrix size = 64 × 64, TR = 2000 ms, TE = 30 ms, flip angle = 70°). For each participant, we recorded 6 runs à 329 functional image volumes, a GRE field map (TE1 = 10.00 ms, TE2 = 12.46 ms), and a T1-weighted structural image (3D MPRAGE, voxel size = 1 mm × 1 mm × 1 mm, FOV = 256 mm × 256 mm, 176 slices, TR = 1900 ms, TE = 2.52 ms, flip angle = 9°). fMRI data were preprocessed and analyzed using SPM12.^2^ Artifacts at the slice-level were corrected using the ArtRepair toolbox (Mazaika et al., 2009). Images were corrected for slice acquisition time differences, realigned and unwarped using the acquired field maps, normalized to MNI space and resliced to 2 mm voxel size using DARTEL, spatially smoothed with a 5 mm full width at half maximum Gaussian kernel, detrended (Macey et al., 2004), and images featuring excessive movement were interpolated (ArtRepair). We fitted a general linear model (GLM, 300 s high-pass filter) to each participant with regressors modeling the stimulations, arm rotations, and catch trials. The first five principal components accounting for the most variance in the cerebrospinal fluid or white matter signal time course each (Behzadi et al., 2007) were added alongside the realignment parameters as regressors of no interest.

First-level contrast images were entered into a group-level flexible factorial design, with the factors condition type (see above), stimulation location (location of the seen touch: top-right or bottom-left), and real arm orientation. Note that the factor stimulation location could also be determined by the location of the felt touch (real index or little finger), which yielded virtually identical results. Activations in the whole brain were assessed for statistical significance applying a voxel-level threshold of *p* < 0.05, family-wise error (FWE) corrected for multiple comparisons. Based on strong prior hypotheses about the well-documented involvement of the left PMv in multisensory comparison and integration, we applied peak FWE-correction within a 10 mm radius spherical region of interest (ROI) centered on coordinates from our previous related study (Limanowski and Blankenburg, 2015; *x* = -38, *y* = 10, *z* = 28, obtained from contrasting synchronous versus asynchronous co-stimulation of a fake and a real arm). For completeness, we report in table format all activations greater than 5 voxels that survived an uncorrected threshold of *p* < 0.001, and we indicate the method used to correct for multiple comparisons. The resulting statistical parametric maps (SPMs) are projected onto the mean normalized structural image. Reported coordinates are in MNI space; the SPM Anatomy toolbox (Eickhoff et al., 2005) was used for anatomical reference. The unthresholded SPMs related to the figures presented here are available online at https://neurovault.org/collections/3393/.

### Behavioral Control Experiment

We conducted an additional behavioral control experiment to verify that the perceptual effects of our stimulation was analogous to typical RHI experiments as published previously. In the fMRI experiment, we were investigating visuotactile integration and comparison processes depending on anatomical congruence of seen and felt touches. This was a novel comparison, and thus differed from the typical control condition used as a contrast to the rubber hand illusion, namely, asynchronous stimulation of the fake and real hand. Although we applied stimulation only for a brief period of time (thus targeting the “pre-illusion” phase associated with the evaluation of multisensory inputs), one could wonder whether the stimulation we used would also reliably induce perceptual differences depending on visuotactile synchrony, and thus be comparable to published studies on the RHI. Therefore, we conducted an additional behavioral control experiment. Eight healthy participants (5 females, mean age = 30.5 years) completed a 4 runs, in between which the real hand’s position was changed between palm and back facing. The 16 stimulation conditions were presented either with synchronous seen and felt touches (as in the fMRI experiment), or with asynchronous seen and felt touches (i.e., with an added delay of 300 ms between the seen and felt touch). We adjusted the stimulation for each participant and verified that the synchrony and asynchrony of stimulations were clearly perceived. As in the fMRI experiment, stimulation blocks were of 6 s duration, with 6 touches presented in a random rhythm.

Further, for any of the fully congruent and synchronous stimulations (i.e., the classical RHI condition) that was rated with at least 2, we included an additional presentation with longer stimulation duration (18 s), and asked the participant to indicate with a verbal response the exact moment at which she would experience the ownership illusion during the stimulation (if she experienced it at all). This verbal report, measured by the experimenter with a stopwatch, was taken as a marker of the illusion onset.

## Results

### Behavioral Results

All participants were able to distinguish whether the real and virtual hands were in congruent or incongruent orientations (mean affirmation rating = 2.75, standard deviation = 0.55; Wilcoxon signed-rank test, *z* = 4.18, *p* < 0.001). Participants also correctly detected 83.75% of the presented catch trials (*SD* = 9.87 %; false alarm rate = 1.41%, *SD* = 2.71%), which was well above chance level (two-tailed *t*-test, *p* < 0.001).

A non-parametric Friedman test revealed significant ownership rating differences between conditions (*χ*^2^ = 41.91, *p* < 0.001). A post-hoc analysis with Wilcoxon signed-rank tests and Bonferroni-corrected alpha levels further showed that ownership ratings were only significantly positive for the fully congruent condition (VT+VP+ vs. zero, *z* = 3.09, *p* < 0.01), which likewise was rated more positively than any other condition (all *z*s > 3.4, all *p*s < 0.001, all CL effect sizes > 0.80). Further, stimulation of the same vs. different fingers was rated significantly more positively even under visuoproprioceptive incongruence (VT+VP- vs. VT-VP-, *z* = 3.55, *p* < 0.001, CL effect size = 0.80).

In a separate behavioral experiment, another group of participants rated the intensity and reported the onset of illusory ownership during the same stimulations, with seen and felt touches delivered either synchronously (as in the main experiment) or asynchronously (with an added delay of 300 ms). The ownership ratings replicated the pattern observed in the ratings of the main experiment: a non-parametric Friedman test revealed significant ownership rating differences between conditions (*χ^2^* = 40.34, *p* < 0.001). A post-hoc analysis with Wilcoxon signed-rank tests showed that ownership ratings of the fully congruent and synchronous condition (VT+VP+_synchronous_, i.e., the classical RHI condition) were significantly higher than any other condition (all *p*s < 0.01, CL effect size = 1). Further, for each of the four conditions, ownership ratings were significantly higher following synchronous versus asynchronous stimulation (all *p*s < 0.05, all CL effect sizes > 0.75). See **Table 1** for details. The mean reported illusion onset in the fully congruent, synchronous condition (the only condition in which the RHI was affirmed by participants) was at 5.44 s (*SD* = 1.46 s) after onset of stimulation.

**Table 1:** Average ownership ratings (with standard deviations in brackets) per condition for the fMRI experiment and the behavioral experiment, with added manipulation of synchrony vs. asynchrony of touches per condition.

	VT+VP+	VT+VP-	VT-VP+	VT-VP-
fMRI experiment	1.55 *(1.36)*	-0.05 *(1.50)*	-0.55 *(1.82)*	-1.90 *(1.29)*
**Behavioral experiment**				
Synchronous touch	1.75 *(1.22)*	-0.38 *(1.64)*	-0.06 *(1.20)*	-0.63 *(1.20)*
Asynchronous touch	0.13 *(1.14)*	-1.91 *(0.63)*	-1.00 *(0.69)*	-1.75 *(0.63)*

### fMRI Results

**FIGURE 2:**
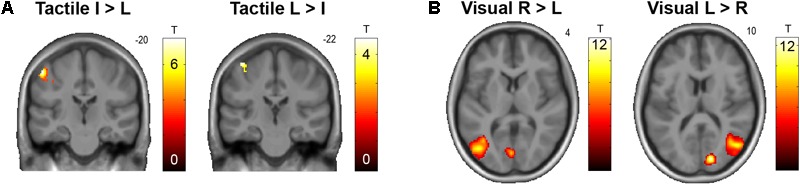
**(A)** Somatotopical organization of responses in the left S1 to tactile stimulation of the right (real) index finger (I) versus little finger (L) and vice versa, across all conditions. The corresponding unthresholded SPM is available at https://neurovault.org/images/59512/. **(B)** Retinotopical organization of responses in the primary visual cortex and motion-sensitive lateral occipitotemporal cortex to visual stimulation in the right (R) versus left (L) hemifield, and vice versa, across all conditions. The corresponding unthresholded SPM is available at https://neurovault.org/images/59513/.

As expected, we observed a clear somatotopical representation of touch locations on the real fingers in the contralateral primary somatosensory cortex (S1, see **Figure 2A**). Contrasting all stimulations on the real index versus little finger revealed an activation cluster at a more lateral inferior location (*x* = -54, *y* = -20, *z* = 56, *T* = 7.47, *p* < 0.05, FWE-corrected); little versus index finger stimulation conversely more dorsally and medially (*x* = -44, *y* = -22, *z* = 68, *T* = 4.54, *p* < 0.001, uncorrected). Visual stimulation in the left versus right visual hemifield was correspondingly retinotopically reflected by significant (*p* < 0.05, corrected) activity increases in the primary and associative visual cortices contralateral to stimulation (**Figure 2B**). Catch trials produced significant activity increases in the bilateral anterior insulae, as well as in fronto-parietal and occipital areas; passive hand rotation produced significant activity increases in a somatomotor network, including the left S1, bilateral secondary somatosensory cortex, and bilateral LOTC.

**FIGURE 3:**
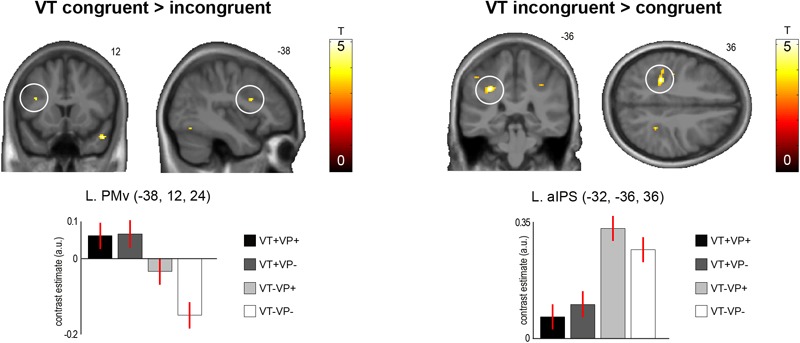
Significant activation differences related to the anatomical congruence of visuotactile stimulation. A region in the left PMv showed significantly stronger responses to touches applied to the same finger of the real and virtual hand (*p* < 0.05, FWE-corrected). Conversely, a region in the anterior part of the left aIPS showed significantly stronger responses to touches applied to different fingers of the two hands (*p* < 0.05, FWE-corrected). These response differences were independent of visuoproprioceptive congruence and spatial (external) visuotactile congruence. The bar plots show the contrast estimates and associated standard errors for each condition at the respective peak voxels. The SPMs are thresholded at *p* < 0.001, uncorrected, for display purposes. See **Table 2** for details. The corresponding unthresholded SPM is available at https://neurovault.org/images/59516/.

In our main analysis, we sought for effects of visuotactile congruence in an anatomical reference frame, i.e., for brain areas that would show response differences to stimulations of the same or different fingers of the real and the virtual hand (**Figure 3** and **Table 2**). Stimulation of the same versus different fingers, i.e., the contrast *(VT+VP+ + VT+VP-) > (VT-VP+ + VT-VP-)*, produced significantly stronger responses in the left PMv (*p* < 0.05, FWE-corrected within an *a priori* defined ROI). The reverse comparison, i.e., *(VT-VP+ + VT-VP-) > (VT+VP+ + VT+VP-)*, revealed significantly stronger responses to stimulation of different versus same fingers at the fundus of the left anterior IPS (aIPS, *p* < 0.05, FWE-corrected), bordering BA 2 of S1. This area of IPS was significantly activated by touch at both somatotopic locations (null conjunction of real index and little finger, *p* < 0.05, FWE-corrected). All of the reported activity differences were consistent over retinotopic and somatotopic stimulation locations, real and virtual hand orientations, and visuoproprioceptive congruence (i.e., we found no significant interaction effects, even when lowering the statistical threshold to *p* < 0.005, uncorrected).

**Table 2:** Activations obtained from the contrasts same vs. different finger stimulation, and vice versa (*p* < 0.001, uncorrected; activations that survived FWE-correction for multiple comparisons are marked in bold font).

Anatomical region	MNI (x, y, z)	Peak T	Peak p (FWE-corr.)
*Same vs. different finger stimulation (VT+VP+ + VT+VT-) > (VT-VP+ + VT-VP-)*			
**L. Ventral premotor cortex**	**-38, 12, 24**	**4.04**	**0.011^∗^**
R. Medial temporal pole	48,10, -26	5.07	–
R. Cingulate	14, -40, 26	4.59	–
R. Supplementary motor area	14, -10, 68	4.39	–
L. Middle frontal gyrus	-36, 30, 44	3.94	–
L. Middle cingulate cortex	8, -18, 32	3.84	–
R. Insula	36, -16, 12	3.83	–
L. Temporal pole	-32, 6, -28	3.62	–
L. Middle temporal gyrus	-44, -44, 0	3.61	–
L. Fusiform gyrus	-38, -66, -14	3.55	–
R. Inferior temporal gyrus	44, -12, -20	3.53	–
R. Insula	34, -14, 22	3.52	–
R. Cingulate	4, -42, 22	3.50	–
R. Superior occipital gyrus	24, -82, 42	3.30	–
*Different vs. same finger stimulation (VT-VP+ + VT-VP-) > (VT+VP+ + VT+VT-)*			
**L. Anterior intraparietal sulcus/BA 2**	**-32, -36, 36**	**5.18**	**0.034**
R. Middle frontal gyrus / dorsal premotor cortex	30, 8, 54	4.35	–
R. Anterior intraparietal sulcus	32, -40, 40	4.30	–
R. Middle frontal gyrus / dorsal premotor cortex	22, -4, 52	4.12	–
R. Middle frontal gyrus / dorsal premotor cortex	52, 16, 44	4.08	–
L./R. Supplementary motor area	2, 18, 48	3.97	–
L. Superior frontal gyrus	-28, 0, 68	3.83	–
R. Postcentral gyrus/intraparietal sulcus	42, -24, 42	3.82	–
L. Inferior parietal lobe	-46, -40, 52	3.74	–
L. Middle frontal gyrus / dorsal premotor cortex	-25, 14, 50	3.64	–
L. Posterior intraparietal sulcus	-22, -64, 40	3.56	–
L. Postcentral gyrus (BA 3a)	-40, -18, 36	3.37	–
L. Insula / secondary somatosensory cortex (OP 3)	-50, -8, 18	3.30	–
L. Cerebellum (Lobule VI)	-8, -76, -24	3.28	–

*^∗^Small volume corrected for multiple comparisons within ROI.*

We did not find any significant activity differences related to visuoproprioceptive congruence, i.e., neither from the main effect nor its interaction with visuotactile congruence.

## Discussion

In this study, we used a somatotopical, virtual reality-based setup to investigate the effects of brief periods of synchronous visuotactile stimulation at anatomically congruent versus incongruent locations (i.e., index or little finger) of a virtual and the real right hand on human brain activity. In contrast to previous work, we aimed to identify BOLD signal correlates of early multisensory comparison processes during the stimulation phase prior to a full-blown RHI experience, and to examine if these comparisons were potentially made with reference to an anatomical (homuncular) reference frame.

fMRI revealed that the left PMv responded significantly more strongly when the touch was seen on the same finger of the virtual hand as it was felt on the real hand. Conversely, the left aIPS responded more strongly when the touch was seen and felt on different fingers. These results suggest fronto-parietal brain areas previously linked to visuotactile integration in PPS (Graziano, 1999; Graziano et al., 2000; Avillac et al., 2007; Gentile et al., 2011) in evaluating visuotactile congruence according to an anatomical reference frame. Interestingly, even following the relatively brief stimulation period, participants reported illusory virtual hand ownership under fully congruent stimulation; these results were replicated in an additional behavioral experiment, which revealed that experienced ownership was significantly higher for fully congruent synchronous versus asynchronous stimulation. Moreover, participants reported higher ownership for anatomically congruent versus incongruent stimulations also under mismatching hand orientations (i.e., visuoproprioceptive incongruence), which demonstrates a perceptual difference depending primarily on visuotactile congruence in an anatomical reference frame. However, these ratings were acquired post scanning and should therefore be interpreted with some caution.

Human brain imaging experiments have demonstrated that, during the RHI, the PMv and IPS integrate spatiotemporally congruent visual and tactile stimuli, within the approximate limits of PPS anchored to the real hand (Ehrsson et al., 2004; Makin et al., 2008; Gentile et al., 2011, 2013; Blanke et al., 2015; Limanowski and Blankenburg, 2015, 2016b). Behavioral studies had shown that the RHI also requires visual and tactile stimuli to be congruent in an anatomical reference frame (Costantini and Haggard, 2007), but the neuronal correlates of this comparison were unknown. Our results provide evidence that the evaluation and potential integration of seen and felt touches based on an anatomical reference frame is implemented in the contralateral PMv and aIPS; they moreover suggest that such an evaluation of visuotactile input occurs very early – potentially before a complete RHI may be induced. This is tentatively supported by the fact that in the behavioral experiment, participants on average reported experiencing the RHI after 5.44 s, which almost covers the entire 6 s stimulation period in the fMRI experiment.

The PMv showed a preference for conditions in which the same finger of the real and virtual hand was stimulated. Our design allowed us to verify that this response pattern was independent of the orientation of the virtual and real hands, and thus independent of the spatial (external) location congruence of the visuotactile stimuli. The PMv is often considered to be the hierarchically highest level of the multisensory body representation targeted by the RHI, among other things based on the fact that activity in this area is often directly related to subjectively perceived body ownership (Ehrsson et al., 2004; Makin et al., 2008; Zeller et al., 2015; Limanowski and Blankenburg, 2016a). Our results support the PMv’s assumed role in assigning body ownership based on a multisensory body and PPS representation, and add novel evidence that the PMv also takes into account the anatomical congruence of visuotactile stimuli when attributing them to one’s body.

Conversely, the left aIPS responded more strongly when touch was seen and felt on different fingers of the real and virtual hand. Again, this response was independent of the hands’ orientation, and of external spatial stimulus location and congruence. The IPS is a multisensory area that compares and aligns visual (external) and tactile (anatomical) reference frames (Bremmer et al., 2001; Graziano and Cooke, 2006; Azañón et al., 2010; cf. Heed et al., 2015), and is a key area involved in various stages of multisensory integration, including evaluation of uncertainty of the individual sensory estimates (Rohe and Noppeney, 2015). Although tactile remapping itself is a fast, sub-second process (Azañón et al., 2010; Heed et al., 2015), it is conceivable that brain areas involved in tactile remapping – and hence having a fundamental role in the processing of touch for multisensory comparisons – may also be involved in determining whether or not some synchronous visuo-tactile input (as during our stimulation) should be integrated. That such computational decisions may also be made within an anatomical reference frame has been suggested before (Haggard et al., 2006; Costantini and Haggard, 2007). Our results suggest that the aIPS of the PPC may be essential to them.

This result may seem surprising, as previous studies have reported increased IPS activation during the RHI (e.g., Ehrsson et al., 2004; Gentile et al., 2013; Limanowski and Blankenburg, 2015). However, we believe our findings may be reconciled with this work. During the RHI, the IPS is most likely involved in integrating the initially mismatching multisensory information and remapping of the corresponding reference frames (Gentile et al., 2013; Limanowski and Blankenburg, 2015), and therefore is specifically engaged during the early stimulation period before illusion onset (Ehrsson et al., 2004; Limanowski and Blankenburg, 2017). The previously observed increased connectivity between the IPS and PMv during the early phase of the RHI (Gentile et al., 2013; Limanowski and Blankenburg, 2015, 2016a) suggests that the IPS communicates with the PMv to enable body ownership, in line with neurocognitive models of the RHI (Makin et al., 2008; Tsakiris, 2010). It may be noteworthy that the IPS activation observed here was located more anteriorly (peak y coordinate = -36, bordering S1) than activations we observed in the IPS during synchronous vs. asynchronous touch (Limanowski and Blankenburg, 2015: *y* = -50; cf. Ehrsson et al., 2004: *y* = -51; Gentile et al., 2013: *y* = -44) or purely visuo-proprioceptive comparisons (Limanowski and Blankenburg, 2017: *y* = -56). There are proposals of functional “somatosensory-to-visual” gradients in the IPS (e.g., Grefkes and Fink, 2005), and an exciting question for future research is whether during the RHI and similar manipulations, multiple comparison processes are in play along such gradients.

In a recent electroencephalography study on the RHI, Zeller et al. (2015) also found increased responses at very similar coordinates to ours (at the junction of IPS and S1) during co-stimulation of a fake hand in an incongruent versus congruent position. In their setup, the incongruent fake hand position implied anatomically incongruent stimulation locations on the real and fake hand—therefore, tentatively, their finding aligns with ours (while we were, moreover, able to isolate the effect of anatomical visuotactile congruence from visuoproprioceptive congruence). Zeller et al. (2015) propose that during congruent touch (the RHI), somatosensory responses are attenuated in S1 and IPS to enable a resolution of multisensory conflict (cf. Limanowski and Blankenburg, 2015). Thus our results could also be interpreted as a relative attenuation of aIPS activity when congruent fingers were seen and felt touched—and conversely, as a prediction error signal when different fingers were seen and felt touched.

In another line of research, the left IPS has also been implied in representing body *structure* (Buxbaum and Coslett, 2001; cf. Haggard et al., 2006; Longo et al., 2010). For example, the left IPS was found activated when participants pointed to different own body parts (versus different spatial locations, Felician et al., 2004), or when they evaluated the distance between body parts (Corradi-Dell’Acqua et al., 2008, 2009). When two fingers of the left and right hand were touched synchronously, the aIPS response increased with structural (i.e., anatomical) distance between the fingers (Rusconi et al., 2009, 2014). In our case, aIPS activity likewise increased with increasing anatomical distance between stimulated fingers—crucially, our visuotactile virtual reality setup allowed us to manipulate the congruence of touches on the fingers of the same hand (rather than touching the invisible left and right hands, cf. Rusconi et al., 2014).

In sum, and in the light of these previous findings, a compelling interpretation of the aIPS activation differences observed in our study is that they reflect an early activation of an anatomical body representation, and perhaps an initial registration of the anatomical mismatch of seen and felt touches in the aIPS, as part of several comparison process of received multisensory information in the IPS.

An alternative interpretation of the observed aIPS responses could be that they reflected vicarious responses to observed touch. Recent research suggests that touches observed on other bodies may be represented on the anatomical map of one’s own body (Thomas et al., 2006), a process that could involve the left aIPS (Ishida et al., 2010, 2015; Chan and Baker, 2015). Note that this interpretation of our findings would also imply a body representation in the aIPS that differentiates between seen and felt touches based on anatomical distance of the touched body parts. Future research will have to test these alternative but potentially complementary explanations against each other, and specifically clarify the role of the IPS in multisensory processes pre- and post-illusory ownership.

Finally, it should be noted that the absence of any significant effects of visuoproprioceptive congruence (i.e., alignment of the hand positions) on brain activity was somewhat unexpected. While it may indeed suggest a general, anatomically based evaluation mechanism, this result contrasts with previous reports and should hence be evaluated by future work—one possibility would be to compare periods of visuoproprioceptive (in)congruence with versus without visuotactile stimulation.

## Conclusion

We found that early anatomical comparisons during visuotactile co-stimulation of a fake and the real hand significantly modulated activity in the PMv and aIPS, brain areas previously linked to visuotactile integration in the PPS and to tactile remapping. These activation differences could not be explained by spatial congruence of the stimuli or by visuoproprioceptive congruence effects, but by visuotactile congruence in an anatomical reference frame. Thus, our results highlight the importance of anatomical congruence for attributing touch and even body parts to oneself, and support the proposal of body representation in multiple reference frames.

## Author Contributions

JL and FB designed the research and wrote the manuscript. JL performed the research and analyzed the data.

## Conflict of Interest Statement

The authors declare that the research was conducted in the absence of any commercial or financial relationships that could be construed as a potential conflict of interest.

## Footnotes

1 http://www.blender.org

2 www.fil.ion.ucl.ac.uk/spm/

## References

[B1] Avillac M. Hamed S. B. Duhamel J. R. (2007). Multisensory integration in the ventral intraparietal area of the macaque monkey. *J. Neurosci.* 27 1922–1932. 10.1523/JNEUROSCI.2646-06.2007 17314288 PMC6673547

[B2] Azañón E. Longo M. R. Soto-Faraco S. Haggard P. (2010). The posterior parietal cortex remaps touch into external space. *Curr. Biol.* 20 1304–1309. 10.1016/j.cub.2010.05.063 20637619

[B3] Behzadi Y. Restom K. Liau J. Liu T. T. (2007). A component based noise correction method (CompCor) for BOLD and perfusion based fMRI. *NeuroImage* 37 90–101. 10.1016/j.neuroimage.2007.04.042 17560126 PMC2214855

[B4] Blanke O. (2012). Multisensory brain mechanisms of bodily self-consciousness. *Nat. Rev. Neurosci.* 13 556–571. 10.1038/nrn3292 22805909

[B5] Blanke O. Slater M. Serino A. (2015). Behavioral, neural, and computational principles of bodily self-consciousness. *Neuron* 88 145–166. 10.1016/j.neuron.2015.09.029 26447578

[B6] Botvinick M. Cohen J. (1998). Rubber hands ‘feel’ touch that eyes see. *Nature* 391:756. 10.1038/35784 9486643

[B7] Bremmer F. Schlack A. Duhamel J. R. Graf W. Fink G. R. (2001). Space coding in primate posterior parietal cortex. *NeuroImage* 14 S46–S51. 10.1006/nimg.2001.0817 11373132

[B8] Brozzoli C. Gentile G. Ehrsson H. H. (2012). That’s near my hand! Parietal and premotor coding of hand-centered space contributes to localization and self-attribution of the hand. *J. Neurosci.* 32 14573–14582. 10.1523/JNEUROSCI.2660-12.2012 23077043 PMC6621451

[B9] Buxbaum L. J. Coslett H. B. (2001). Specialised structural descriptions for human body parts: evidence from autotopagnosia. *Cogn. Neuropsychol.* 18 289–306. 10.1080/02643290126172 20945217

[B10] Chan A. W. Y. Baker C. I. (2015). Seeing is not feeling: posterior parietal but not somatosensory cortex engagement during touch observation. *J. Neurosci.* 35 1468–1480. 10.1523/JNEUROSCI.3621-14.2015 25632124 PMC4308594

[B11] Corradi-Dell’Acqua C. Hesse M. D. Rumiati R. I. Fink G. R. (2008). Where is a nose with respect to a foot? The left posterior parietal cortex processes spatial relationships among body parts. *Cereb. Cortex* 18 2879–2890. 10.1093/cercor/bhn046 18424775

[B12] Corradi-Dell’Acqua C. Tomasino B. Fink G. R. (2009). What is the position of an arm relative to the body? Neural correlates of body schema and body structural description. *J. Neurosci.* 29 4162–4171. 10.1523/JNEUROSCI.4861-08.2009 19339611 PMC6665372

[B13] Costantini M. Haggard P. (2007). The rubber hand illusion: sensitivity and reference frame for body ownership. *Conscious. Cogn.* 16 229–240. 10.1016/j.concog.2007.01.001 17317221

[B14] Ehrsson H. H. (2012). “The concept of body ownership and its relation to multisensory integration,” in *The New Handbook of Multisensory Processes* ed. Stein B. E. (Cambridge, MA: MIT Press).

[B15] Ehrsson H. H. Spence C. Passingham R. E. (2004). That’s my hand! Activity in premotor cortex reflects feeling of ownership of a limb. *Science* 305 875–877. 10.1126/science.1097011 15232072

[B16] Eickhoff S. B. Stephan K. E. Mohlberg H. Grefkes C. Fink G. R. Amunts K. (2005). A new SPM toolbox for combining probabilistic cytoarchitectonic maps and functional imaging data. *NeuroImage* 25 1325–1335. 10.1016/j.neuroimage.2004.12.034 15850749

[B17] Felician O. Romaiguère P. Anton J. L. Nazarian B. Roth M. Poncet M. (2004). The role of human left superior parietal lobule in body part localization. *Ann. Neurol.* 55 749–751. 10.1002/ana.20109 15122719

[B18] Gentile G. Guterstam A. Brozzoli C. Ehrsson H. H. (2013). Disintegration of multisensory signals from the real hand reduces default limb self-attribution: an fMRI study. *J. Neurosci.* 33 13350–13366. 10.1523/JNEUROSCI.1363-13.2013 23946393 PMC3742923

[B19] Gentile G. Petkova V. I. Ehrsson H. H. (2011). Integration of visual and tactile signals from the hand in the human brain: an fMRI study. *J. Neurophysiol.* 105 910–922. 10.1152/jn.00840.2010 21148091 PMC3059180

[B20] Graziano M. S. (1999). Where is my arm? The relative role of vision and proprioception in the neuronal representation of limb position. *Proc. Natl. Acad. Sci. U.S.A.* 96 10418–10421. 10.1073/pnas.96.18.10418 10468623 PMC17903

[B21] Graziano M. S. Cooke D. F. (2006). Parieto-frontal interactions, personal space, and defensive behavior. *Neuropsychologia* 44 845–859. 10.1016/j.neuropsychologia.2005.09.009 16277998

[B22] Graziano M. S. Cooke D. F. Taylor C. S. (2000). Coding the location of the arm by sight. *Science* 290 1782–1786. 10.1126/science.290.5497.1782 11099420

[B23] Graziano M. S. Yap G. S. Gross C. G. (1994). Coding of visual space by premotor neurons. *Science* 266 1054–1057. 10.1126/science.7973661 7973661

[B24] Grefkes C. Fink G. R. (2005). The functional organization of the intraparietal sulcus in humans and monkeys. *J. Anat.* 207 3–17. 10.1111/j.1469-7580.2005.00426.x 16011542 PMC1571496

[B25] Haggard P. Kitadono K. Press C. Taylor-Clarke M. (2006). The brain’s fingers and hands. *Exp. Brain Res.* 172 94–102. 10.1007/s00221-005-0311-8 16369787

[B26] Heed T. Buchholz V. N. Engel A. K. Röder B. (2015). Tactile remapping: from coordinate transformation to integration in sensorimotor processing. *Trends Cogn. Sci.* 19 251–258. 10.1016/j.tics.2015.03.001 25843541

[B27] Ishida H. Nakajima K. Inase M. Murata A. (2010). Shared mapping of own and others’ bodies in visuotactile bimodal area of monkey parietal cortex. *J. Cogn. Neurosci.* 22 83–96. 10.1162/jocn.2009.21185 19199418

[B28] Ishida H. Suzuki K. Grandi L. C. (2015). Predictive coding accounts of shared representations in parieto-insular networks. *Neuropsychologia* 70 442–454. 10.1016/j.neuropsychologia.2014.10.020 25447372

[B29] Kilteni K. Maselli A. Koerding K. Slater M. (2015). Over my fake body: body ownership illusions for studying the multisensory basis of own-body perception. *Front. Hum. Neurosci.* 9:141. 10.3389/fnhum.2015.00141 25852524 PMC4371812

[B30] Limanowski J. Blankenburg F. (2015). Network activity underlying the illusory self-attribution of a dummy arm. *Hum. Brain Mapp.* 36 2284–2304. 10.1002/hbm.22770 25708317 PMC6869824

[B31] Limanowski J. Blankenburg F. (2016a). Integration of visual and proprioceptive limb position information in human posterior parietal, premotor, and extrastriate cortex. *J. Neurosci.* 36 2582–2589. 10.1523/JNEUROSCI.3987-15.2016 26937000 PMC6604875

[B32] Limanowski J. Blankenburg F. (2016b). That’s not quite me: limb ownership encoding in the brain. *Soc. Cogn. Affect. Neurosci.* 11 1130–1140. 10.1093/scan/nsv079 26089343 PMC4927034

[B33] Limanowski J. Blankenburg F. (2017). Posterior parietal cortex evaluates visuoproprioceptive congruence based on brief visual information. *Sci. Rep.* 7:16659. 10.1038/s41598-017-16848-7 29192256 PMC5709509

[B34] Longo M. R. Azañón E. Haggard P. (2010). More than skin deep: body representation beyond primary somatosensory cortex. *Neuropsychologia* 48 655–668. 10.1016/j.neuropsychologia.2009.08.022 19720070

[B35] Macaluso E. Driver J. (2005). Multisensory spatial interactions: a window onto functional integration in the human brain. *Trends Neurosci.* 28 264–271. 10.1016/j.tins.2005.03.008 15866201

[B36] Macey P. M. Macey K. E. Kumar R. Harper R. M. (2004). A method for removal of global effects from fMRI time series. *NeuroImage* 22 360–366. 10.1016/j.neuroimage.2003.12.042 15110027

[B37] Makin T. R. Holmes N. P. Ehrsson H. H. (2008). On the other hand: dummy hands and peripersonal space. *Behav. Brain Res.* 191 1–10. 10.1016/j.bbr.2008.02.041 18423906

[B38] Makin T. R. Holmes N. P. Zohary E. (2007). Is that near my hand? Multisensory representation of peripersonal space in human intraparietal sulcus. *J. Neurosci.* 27 731–740. 10.1523/JNEUROSCI.3653-06.2007 17251412 PMC6672897

[B39] Mazaika P. Hoeft F. Glover G. H. Reiss A. L. (2009). “Methods and software for fMRI analysis for clinical subjects,” in *Proceedings of the Annual Meeting of the Organization for Human Brain Mapping* (San Francisco, CA). 10.1016/S1053-8119(09)70238-1

[B40] Meredith M. A. Stein B. E. (1986). Visual, auditory, and somatosensory convergence on cells in superior colliculus results in multisensory integration. *J. Neurophysiol.* 56 640–662. 10.1152/jn.1986.56.3.640 3537225

[B41] Rizzolatti G. Fadiga L. Fogassi L. Gallese V. (1997). The space around us. *Science* 277 190–191. 10.1126/science.277.5323.190 9235632

[B42] Rohe T. Noppeney U. (2015). Cortical hierarchies perform Bayesian causal inference in multisensory perception. *PLoS Biol.* 13:e1002073. 10.1371/journal.pbio.1002073 25710328 PMC4339735

[B43] Rusconi E. Gonzaga M. Adriani M. Braun C. Haggard P. (2009). Know thyself: behavioral evidence for a structural representation of the human body. *PLoS One* 4:e5418. 10.1371/journal.pone.0005418 19412538 PMC2671600

[B44] Rusconi E. Tame L. Furlan M. Haggard P. Demarchi G. Adriani M. (2014). Neural correlates of finger gnosis. *J. Neurosci.* 34 9012–9023. 10.1523/JNEUROSCI.3119-13.2014 24990921 PMC6608250

[B45] Samad M. Chung A. J. Shams L. (2015). Perception of body ownership is driven by Bayesian sensory inference. *PLoS One* 10:e0117178. 10.1371/journal.pone.0117178 25658822 PMC4320053

[B46] Thomas R. Press C. Haggard P. (2006). Shared representations in body perception. *Acta Psychol.* 121 317–330. 10.1016/j.actpsy.2005.08.002 16194527

[B47] Tsakiris M. (2010). My body in the brain: a neurocognitive model of body-ownership. *Neuropsychologia* 48 703–712. 10.1016/j.neuropsychologia.2009.09.034 19819247

[B48] Wuensch K. L. (2015). *Nonparametric Effect Size Estimators.* Available at: http://core.ecu.edu/psyc/wuenschk/docs30/Nonparametric-EffectSize.pdf [accessed June 16, 2015].

[B49] Yamamoto S. Kitazawa S. (2001). Reversal of subjective temporal order due to arm crossing. *Nat. Neurosci.* 4 759–765. 10.1038/89559 11426234

[B50] Zeller D. Litvak V. Friston K. J. Classen J. (2015). Sensory processing and the rubber hand illusion—an evoked potentials study. *J. Cogn. Neurosci.* 27 573–582. 10.1162/jocn_a_00705 25170795

